# Accessibility of Urgent Care Centers: A Socioeconomic and Geospatial Evaluation

**DOI:** 10.5811/westjem.35583

**Published:** 2025-09

**Authors:** Parnika Telagi, Richard Sadler, Praval Telagi, Kevin McGurk

**Affiliations:** * Medical College of Wisconsin, Milwaukee, Wisconsin; † University of Illinois, Department of Computer Science, Urbana, Illinois; ‡ Michigan State University College of Human Medicine, Department of Public Health and Family Medicine, East Lansing, Michigan; § Medical College of Wisconsin, Department of Emergency Medicine, Milwaukee, Wisconsin

## Abstract

**Introduction:**

Urgent care centers (UC) play an important role in addressing non-emergent health concerns, offering a convenient alternative to emergency departments (ED). However, accessibility to UCs can vary based on transportation availability and socioeconomic factors. In this study we evaluated the geospatial accessibility of UCs and EDs in Milwaukee County, Wisconsin, and sought to characterize the relationship between transit options, socioeconomic vulnerability, and access to care.

**Methods:**

We included 13 EDs and 13 UCs in the study. Public and private transit times between census tracts in Milwaukee County and the nearest UC or ED were calculated using an application programming interface that recorded data from Google Maps. We employed socioeconomic vulnerability index (SEVI) scores to define community vulnerability. Statistical analyses, including Mann-Whitney U tests and Pearson correlation coefficients, were used to determine differences in commute times and their relationship with socioeconomic status.

**Results:**

Private transit times were shorter than public transit times when commuting to the nearest ED (7 minutes vs 22 minutes, *P* <.001*)* and the nearest UC (9 minutes vs 31 minutes, *P* < .001*)*. The EDs were generally more accessible than UCs, with shorter transit (22 vs 31 minutes, *P* < .001) and walk times (11 vs 14 minutes, *P* <.001). Socioeconomically disadvantaged communities with higher SEVI scores had longer private transit times to UCs (*r* = 0.17, P = .003) while having shorter public transit times to EDs (*r* = −.21, *P* < .001).

**Conclusion:**

Access to urgent care centers and EDs in Milwaukee County is influenced by socioeconomic factors and transportation modes. While EDs are more accessible to socioeconomically vulnerable communities, UCs are less accessible, which may contribute to higher ED utilization for non-emergent needs. These findings highlight the need to address transportation limitations as a social determinant of health that can impact how disadvantaged populations seek care and the implications for non-emergent ED use and ED crowding.

## INTRODUCTION

Urgent care centers (UC) play an increasingly important role in delivering expedited care to patients with non-emergent health concerns. Utilization has steadily increased in the US since their advent in the 1970s and growth through the 2000s, with more than 29% of adults having at least one UC visit annually.^1^–^4^ While no consensus definition exists for what constitutes an UC, these facilities are generally capable of caring for patients with mild injuries and ailments, do not require appointments, and provide service over extended business hours.^5^,^6^ Urgent care centers are often viewed by patients as an appealing alternative to emergency department (ED) or primary care physician (PCP) visits for reasons of cost, convenience, or both.^7^,^8^ As many will ultimately seek ED care for non-emergent needs, redirecting these cases to UCs has the potential to confer savings to the healthcare system.^6^

The choice of where to pursue care for non-emergent healthcare needs is impacted by a multitude of factors including insurance, timing, access to a PCP, socioeconomic status and available transportation.^9^ Transportation represents an essential social determinant of health and can affect how patients access healthcare and the facilities they visit.^10^–^14^ Regardless of the presence of reliable or timely transportation, patients may prioritize geographic proximity over other factors when choosing a location for their care.^15^ Patients may use public or private transportation to visit UCs and EDs. When neither is appropriate or accessible, individuals may also use emergency medical services (EMS) to go to the ED.

Milwaukee County is the most populous county in Wisconsin and home to nearly one million residents in a geographically and socioeconomically diverse area. The demographic composition of its population is 51% White, 26% Black, 18% Hispanic/Latino, 10% multiracial, 5% Asian, and 8% other races. Within the county, 14% of families live below the poverty line, and unemployment rates exceed state and national averages. Approximately 60% of the county’s population resides within the City of Milwaukee. The remainder of Milwaukee County is comprised of a mix of suburban areas and a few outlying rural communities.^16^

Public transportation within the county includes bus lines and limited streetcar services. While UCs have previously been associated with urban areas, higher income levels and a greater prevalence of private insurance, the accessibility of these facilities via different modes of transit is generally unknown. Given the expanding role that UCs play and the importance of transportation on how and when patients seek medical care, we sought to quantify the accessibility of UCs by public and private transit and to characterize the association between accessibility and the socioeconomic status of communities.

## METHODS

While many facilities advertise as UCs, widely disparate clinical and diagnostic capabilities exist across these centers. As no uniformly accepted definition exists, we employed criteria in accordance with Urgent Care Association of America standards and consistent with previously published research.^17^,^18^ These criteria include extended hours of operation, weekend availability, the option to be seen without an appointment, basic lab and imaging capabilities, and the ability to perform minor procedures.

We performed statistical analyses using R 4.2.3 (The R Foundation for Statistical Computing, Vienna, Austria).^19^ Group differences were calculated using the Mann-Whitney U test. We calculated the relationship between socioeconomic vulnerability index (SEVI) scores and transit times using the Pearson correlation coefficient. We used ArcMap 10.8 (Environmental Systems Research Institute, Inc, Redlands, CA) to combine all spatial data, including the location of UCs and EDs, travel time to UCs and EDs by census block, Social Vulnerability Index (SVI) and SEVI scores, and the boundaries of Milwaukee County and the city of Milwaukee.

**Population Health Research Capsule**

What do we already know about this issue?

*Emergency department (ED) visits have increased for non-emergent issues that could otherwise be seen at an urgent care center (UC). Transportation often impacts how patients seek care*.

What was the research question?

*Do commute times to the nearest ED and UC differ, and is there an association with a community’s socioeconomic standing?*

What was the major finding of the study?

*Commute times to EDs were shorter than UCs via public transit (22 vs 31 minutes, P < .001) and private transit (7 vs 9 minutes, P < .001) and were correlated with socioeconomic status as poorer areas had longer private commutes to UCs (r = .17, P = .003) and shorter public commutes to EDs (r =* −*.21, P < .001)*.

How does this improve population health?

*Transit times and geographic proximity to the nearest ED or UC are associated with socioeconomic status and may influence how and where patients seek care*.

All EDs and UCs meeting inclusion criteria within Milwaukee County were included for analysis. We sought to address potential errors introduced by the edge effect, a phenomenon by which spatial error is introduced when features outside a study area that individuals are likely to visit are excluded from analysis (typically leading to artificially poorer calculated access). To do so, we also included facilities outside Milwaukee County if they were the closest care center by transit time, generally for communities on or near the county’s border.^20^,^21^

We used publicly available data online from the US Census Bureau, Centers for Disease Control and Prevention (CDC), and Agency for Toxic Substances and Disease Registry to characterize the census tracts in Milwaukee County and their relative social vulnerability as measured by the SVI. This index has further subclassifications grouped by theme including a summation of socioeconomic factors we have abbreviated as the SEVI. The SEVI includes factors such as poverty, unemployment, levels of education, and lack of health insurance. The SEVI ranges from 0 to 1, with higher values indicating increased socioeconomic vulnerability.

We used Google Maps to compute the public and private transit information between each census tract to its nearest UC and ED. We wrote a program using the Google Maps application programming interface to collect the data, which compared transit times between each census tract to each UC and ED, saving the shortest times. For public transit information, we computed and recorded total transit time, number of bus transfers, and walking time, Transit times were computed over multiple days and times, including mornings, evenings, weekdays, and weekends. While the data presented represents transit at 8 am on a Monday, the statistical trends were unchanged across all time points assessed.

This study was declared exempt by the Medical College of Wisconsin Institutional Review Board.

## RESULTS

We included 13 EDs and 13 UCs for analysis (10 each inside Milwaukee County and three each just outside its borders). Median commute times to the closest ED and UC using public and private transit are shown below (Table). The Mann-Whitney U test results shown compare transit times to the nearest UC vs the nearest ED.Private transit times were faster than public transit times when commuting to the nearest ED (7 minutes vs 22 minutes, *P* <.001*)* and the nearest UC (9 minutes vs 31 minutes, *P* < .001*)*. The longest private and public transit times to the nearest ED were 16 and 77 minutes, respectively. The longest private and public transit times to the nearest UC were 15 and 62 minutes, respectively. When comparing commutes to the nearest ED vs UC, private transit time, public transit time, and total minutes of walking were all shorter for EDs than UCs. We found no statistically significant difference in the number of bus transfers needed to travel to the nearest UC vs ED.

**Figure 1:**
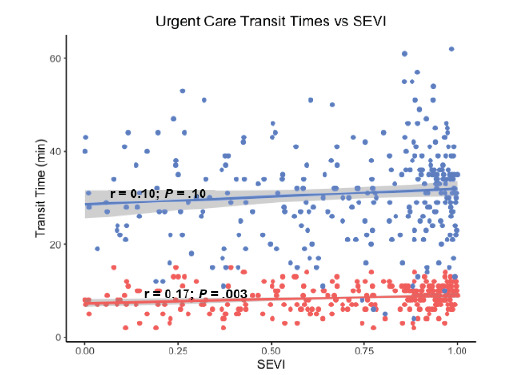
Association between socioeconomic vulnerability and transit times to the nearest urgent care. *Note that blue indicates public transit and red indicates private transit*. *SEVI*, Socioeconomic Vulnerability Index.

**Figure 2:**
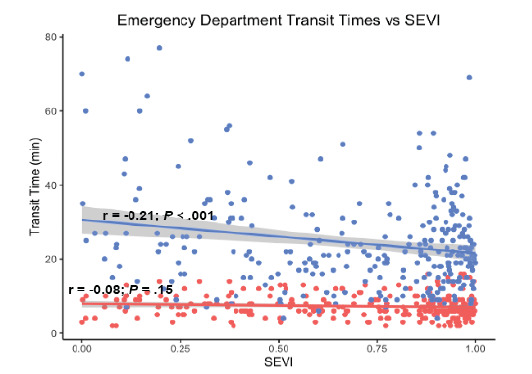
Association between socioeconomic vulnerability and transit times to the nearest emergency department *Note that blue indicates public transit and red indicates private transit*. *SEVI*, Socioeconomic Vulnerability Index. *UC*, urgent care center; *ED*, emergency department.

Socioeconomic vulnerability was associated with increased public and private transit times to the nearest UC (*P =* .10 *and P* = .003, respectively) as shown in Figure 1. Conversely, SEVI score was associated with decreased public and private transit times to the nearest ED (*P* < .001 and *P* = .15, respectively) as shown in Figure 2.

**Figure 3:**
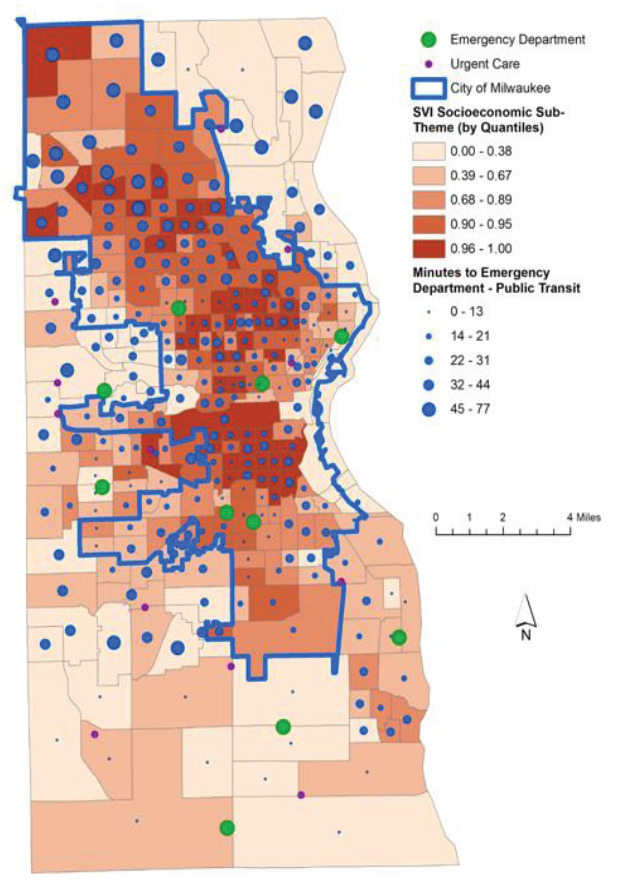
Map of Milwaukee County displaying socioeconomic vulnerability transit times to healthcare facilities by public transportation. *SVI*, Social Vulnerability Index,

**Figure 4:**
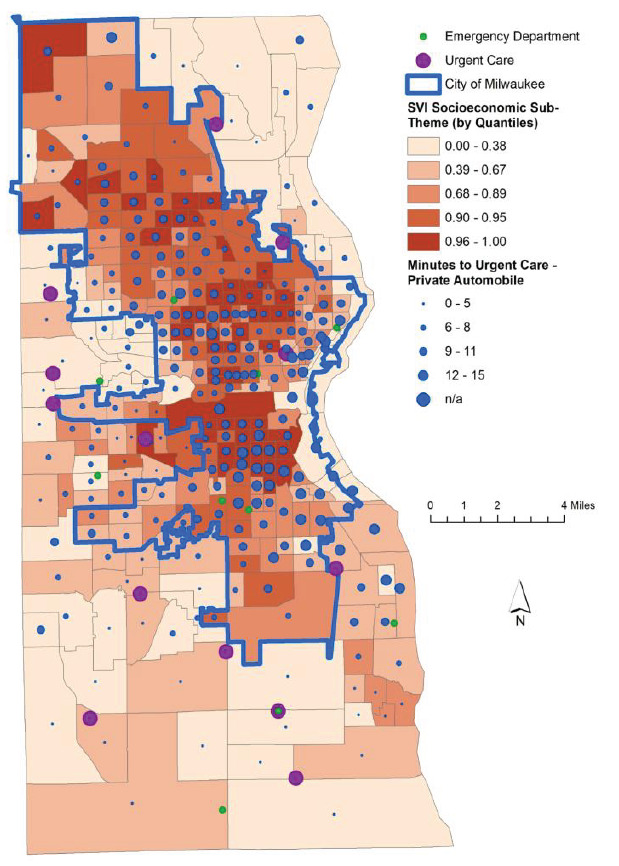
Map of Milwaukee County displaying socioeconomic vulnerability and private transit times to healthcare facilities by private vehicle. *SVI*, Social Vulnerability Index.

The relationship between the SEVI score of each census tract and its proximity to the nearest ED is well represented using a map of Milwaukee County (Figure 3). The length of commute from each tract to the nearest ED corresponds to the size of the blue dot within that municipality. The most socioeconomically vulnerable communities grouped by quintile are shown to have shorter commutes to the nearest ED as compared to wealthier census tracts. Commuting to UCs shows the opposite relationship. When comparing private transit times to the nearest UC, the most vulnerable census tracts have longer commute times in general than less disadvantaged communities (Figure 4).

## DISCUSSION

Unsurprisingly, our study demonstrated that private transit times were shorter than public transportation to both UCs and EDs. While no commute via private transit took longer than 16 minutes, in certain communities public transit took as long as 77 minutes and frequently required multiple bus transfers and lengthy walks. This is particularly impactful when considering patients with disabilities or impaired mobility for whom lengthy commute or walk times may be untenable.

Importantly, we found disparate commute times to both types of healthcare facilities when compared to the socioeconomic vulnerability of each census tract. Socioeconomically disadvantaged communities had a statistically significant increase in private transit times to UCs and a decrease in public transit times to the nearest EDs. The general trend for all modes of transport was that individuals in more affluent communities had easier access to UCs, whereas EDs were more accessible to patients from poorer communities. These results mirror some of the findings from prior research investigating the accessibility of hospital-based vs freestanding EDs in some large metropolitan areas.^22^ The freestanding EDs, like the UCs in our study, were less accessible by public transportation as compared to hospital-based EDs and were generally located in more affluent areas.

As EDs deal with a sustained crowding crisis, minimizing use for non-emergent complaints has generally been viewed as an important mitigation step.^23^,^24^ In particular, medical concerns that could be adequately addressed in alternative care settings have been the focus of substantial study.^9^,^25^ While primary care represents an important alternative, resource limitations also impact the availability of expedient appointments even for those with established PCPs.^26^–^29^ Thus, UCs are often viewed as another release valve for ED crowding with respect to the care of patients with minor injuries and ailments. However, the extent to which UCs might help to alleviate ED crowding is likely tempered in communities with limited access to these centers.

Decisions on where to seek care can be complex, and the influences are multifactorial. Urgent care centers are not beholden to the same federal care mandates, and the increasing number of UCs and decreasing number of hospitals nationwide is testament to the current financial, legal, and regulatory climate for US healthcare.^30^,^31^ While treatment in the ED is regarded as a more expensive mode of healthcare delivery than UCs, this may not be true at the individual level when accounting for considerations such as insurance status and visit copays.^32^ How and why patients choose a particular type of healthcare facility for non-emergent needs remains an important field of inquiry, and this study adds valuable context to a body of literature that also examines novel approaches to reduce low-acuity ED visits.^33^,^34^ Our findings indicate that when evaluating where patients seek care, geographic proximity and public transit accessibility should be considered. Socioeconomic status and transit times to EDs and UCs were significantly correlated, which may asymmetrically impact communities with higher socioeconomic vulnerability. The ease of access or lack thereof to UCs has implications for both ED use for non-emergent concerns and patterns of EMS use.

## LIMITATIONS

This study measured the accessibility of healthcare facilities in one county in the state of Wisconsin. While Milwaukee is geographically and socioeconomically diverse, it is not necessarily nationally representative. As population density and public transit infrastructure differ in other regions, the geospatial considerations related to healthcare access may also change. Additionally, different states and municipalities may have statutes or regulations that directly or indirectly influence the location of these care facilities. Lastly, while this study examined transit times to UCs, proximity to care does not necessarily ensure accessibility. Variable UC payment models and upfront patient costs could affect the pattern of use independent of transit times, particularly for socioeconomically vulnerable populations.

## CONCLUSION

Commute times to the nearest EDs and urgent care centers via public or private transit were associated with the socioeconomic vulnerability of census tracts in Milwaukee County. Further study is needed to determine the impact of this disparity in transit time on healthcare use for non-emergent medical needs.

## Figures and Tables

**Table:** Median commute times to the closest emergency department and urgent care using public and private transit.

	Public Transit		Private Transit
		
	Median Total Transit Time	Median Walk Time	Median Total Transit Time
UC	31	14	9
ED	22	11	7
Mann-Whitney U test	*P* < .001	*P* < .001	*P* < .001
